# HSF1 Attenuates the Release of Inflammatory Cytokines Induced by Lipopolysaccharide through Transcriptional Regulation of *Atg10*

**DOI:** 10.1128/spectrum.03059-22

**Published:** 2023-01-04

**Authors:** Hong Tan, Feifei Huang, Meiyuan Huang, Xia Wu, Zhongyi Tong

**Affiliations:** a Department of Pathology, The Second Xiangya Hospital, Central South University, Changsha, Hunan, China; b Department of Pathology, Shenzhen People’s Hospital, Second Clinical Medical College of Jinan University, Shenzhen, Guangdong, China; c Department of Pathology, Zhuzhou Hospital Affiliated to Xiangya School of Medicine, Central South University, Zhuzhou, Hunan, China; Institute of Microbiology, Chinese Academy of Sciences

**Keywords:** heat shock factor 1 (HSF1), inflammatory cytokines, lipopolysaccharide (LPS), endotoxemia, autophagy related gene 10 (*Atg10*)

## Abstract

Autophagy plays an important role in endotoxemic mice, and heat shock factor 1 (HSF1) plays a crucial protective role in endotoxemic mice. However, the protective mechanisms of HSF1 are poorly understood. In this text, bioinformatics analysis, chromatin immunoprecipitation, and electrophoresis mobility shift assay were employed to investigate the underlying mechanisms. The results showed that the release of inflammatory cytokines increased and autophagy decreased significantly in *Hsf1^−/−^* endotoxemic mice compared with those in *Hsf1^+/+^* endotoxemic mice. HSF1 could directly bind to the noncoding promoter region of the autophagy-related gene 10 (*Atg10*). The expression of ATG10 and the ratio of LC3-II/LC3-I were obviously decreased in LPS-treated *Hsf1*^−/−^ peritoneal macrophages (PM) versus those in LPS-treated *Hsf1*^+/+^ PM. Overexpression of HSF1 increased the level of the ATG10 protein and enhanced the ratio of LC3-II/LC3-I in RAW264.7 cells. In contrast, silencing of HSF1 decreased the expression of ATG10 and markedly lowered the ratio of LC3-II/LC3-I. In a cotransfected cell experiment, the upregulation of autophagy by overexpression HSF1 was reversed by small interfering RNA (siRNA)-ATG10. Compared with the overexpression HSF1, the release of inflammatory cytokines induced by lipopolysaccharide (LPS) was decreased in pcDNA3.1-HSF1 with siRNA-ATG10 cotransfected RAW264.7 cells. On the other hand, the decrease of autophagy by siRNA-HSF1 was compensated by overexpression of ATG10. Compared with siRNA-HSF1, the release of inflammatory cytokines induced by LPS was increased in siRNA-HSF1 with pcDNA3.1-ATG10 cotransfected RAW264.7 cells. These results presented a novel mechanism that HSF1 attenuated the release of inflammatory cytokines induced by LPS through transcriptional regulation of *Atg10*. Targeting of HSF1-*Atg10*-autophagy might be an attractive strategy in endotoxemia therapeutics.

**IMPORTANCE** HSF1 plays an important protective role in endotoxemic mice. However, the protective mechanisms of HSF1 are poorly understood. In the present study, we demonstrated that HSF1 upregulated ATG10 through specifically binding *Atg10* promoter’s noncoding region in LPS-treated PM and RAW264.7 cells. By depletion of HSF1, the expression of ATG10 was significantly decreased, leading to aggravate releasing of inflammatory cytokines in LPS-treated RAW264.7 cells. These findings provided a new mechanism of HSF1 in endotoxemic mice.

## INTRODUCTION

Sepsis, a kind of inflammation caused by infection, can develop into life-threatening bacteremia and bacterial-driven endotoxemia (1). Most of the therapeutics targeting sepsis have failed in the clinics (2). We are in an urgent need for a multitargeting therapeutic approach for sepsis.

Lipopolysaccharide (LPS) is a component of bacteria that can induce immunogenic responses, which results in advanced sepsis in organs after the exposure of immune cells to such bacterial components (3, 4). Endotoxemia caused by LPS is marked by whole-body activation of the inflammatory responses, which can lead to multiple-organ failure and shock (5). A response to endotoxemia is the release of proinflammatory cytokines from its main source, visceral adipose tissue, which is associated with disease mortality (6, 7). Thus, attenuating the release of proinflammatory cytokines may be a crucial strategy in the treatment of endotoxemia.

As a transcription factor of heat shock proteins (HSPs), heat shock factor 1 (HSF1) plays a key role in combating endotoxemia, including inhibition of proinflammatory cytokine genes, such as tumor necrosis factor-α (TNF-α), interleukin 1 (IL-1), and IL-6 (8, 9). HSF1 can bind directly to the promoter of these inflammatory cytokines. Previous studies have shown that HSF1 inhibits the expression of TNF-α through direct binding to the TNF-α promoter (10). HSF1 represses the expression of IL-1β through physical interaction with the nuclear factor for IL-6 (NF-IL-6), which is an activator of IL-1β (11). HSF1 inhibits the expression of IL-6 by inducing the activation of transcription factor 3 (ATF3) (12), a negative regulator of inflammatory cytokines, including IL-6 (13).

HSF1 also reduces systemic markers of organ dysfunction, including alanine aminotransferase, aspartate aminotransferase, lactate dehydrogenase, and blood urea nitrogen (14). Studies indicate that HSF1 is essential for the prevention of systemic inflammation and tissue damage caused by bacterial endotoxins or sepsis. HSF1 could negatively regulate HMGB1 and be involved in regulating asthma (15). HSF1 plays an indispensable role in the initiation, promotion, and progression of cancer (16). Therefore, HSF1 operates in a multifaceted manner, expanding far beyond the heat shock response (HSR).

Autophagy is a dynamic process of a cell where biomacromolecules and damaged organelles are engulfed in an autophagic vesicle that merges with a lysosome and forms an autolysosome. Autolysosomes degrade the contents of the vesicle, such as organelles and proteins, through a variety of bioenzymes from the lysosome (17). As a cytoplasmic degradation pathway, autophagy protects cells from both exogenous hazards, such as infection and endogenous inflammation. In any type of cells, the cytoplasmic cleansing function of autophagy is by default anti-inflammatory and capable of activating cell-autonomous inflammatory responses (18, 19). Autophagy or individual autophagy factors target key intracellular combinations and platforms of proinflammatory signaling that inhibit or degrade to limit inflammation (20). In conclusion, autophagy plays an important role in inflammation.

In the previous study, HSF1 Thr120 phosphorylation promoted proteostasis and carboplatin-induced autophagy in breast tumor progression (21). Heat treatment resulted in an elevation in pAMPK (T172), Beclin-1, and LC3 II and an upregulation of HSF1 (22). However, the mechanisms of HSF1 in the process of autophagy remain to be fully characterized. In this paper, bioinformatics, chromatin immunoprecipitation assay, electrophoretic mobility shift assay, and other methods were used to explore the mechanisms of HSF1 in regulating autophagy. Our findings will benefit the development of new treatment strategies for endotoxemia.

## RESULTS

### HSF1 attenuated the release of inflammatory cytokines, which was associated with autophagy in endotoxemic mice.

**FIG 1:**
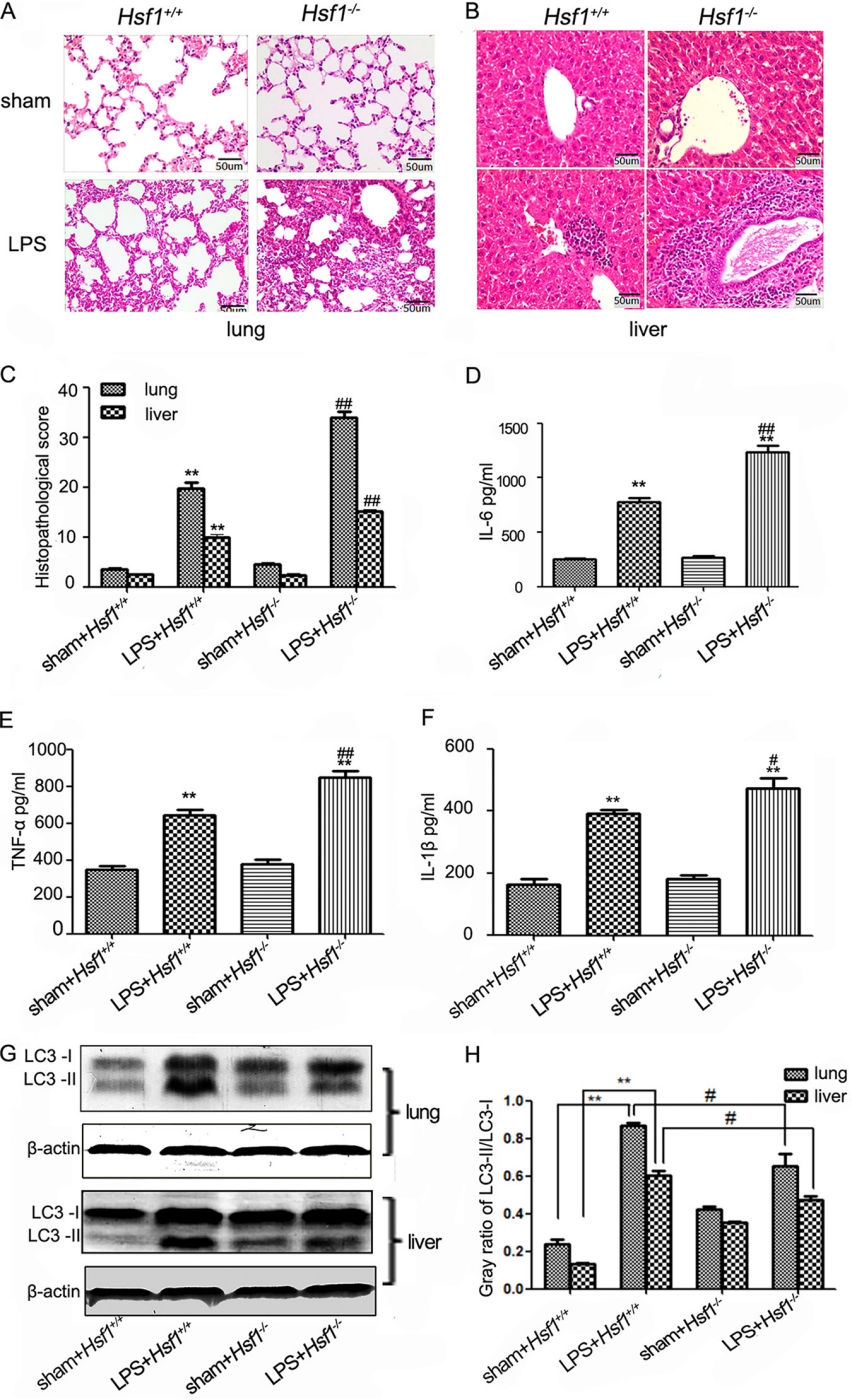
HSF1-mediated attenuating the release of inflammatory cytokines was associated with autophagy. (A and B) The histomorphology in the lung (scale bar, 50 μm) and liver (scale bar, 50 μm) of *Hsf1^+/+^* and *Hsf1^−/−^* endotoxemic mice by H&E staining. (C) Semiquantitative analysis about the histopathological injury of lung and livers were evaluated. **, *P < *0.01 versus *Hsf1^+/+^* sham mice (*n* = 6); **^##^**, *P < *0.01 versus *Hsf1^+/+^* LPS mice (*n* = 6). (D to F) The concentrations of IL-6, TNF-α, and IL-1β were detected in *Hsf1^+/+^* and *Hsf1^−/−^* endotoxemic mice serum. **, *P < *0.01 versus *Hsf1^+/+^* sham mice (*n* = 6); **^#^**, *P < *0.05; **^##^**, *P < *0.01 versus *Hsf1^+/+^* LPS mice (*n* = 6). (G and H) Mice were treated with LPS for 12 h, and the expressions of LC3-II and LC3-I protein were measured with anti-LC3 antibody in the liver and lung; **, *P < *0.01 versus *Hsf1^+/+^* sham mice (*n* = 6); **^#^** *P < *0.05 versus *Hsf1^+/+^* LPS mice (*n* = 6).

First, *Hsf1^+/+^* and *Hsf1^−/−^* endotoxemic mice were established by intraperitoneal injection of LPS, and the histomorphology of the liver and lung in mice was examined by hematoxylin and eosin (H&E) staining. As shown in Fig. 1A to C, visual damages of organs were more severe in *Hsf1^−/−^* endotoxemic mice than those in *Hsf1^+/+^* endotoxemic mice. The symptoms included a large amount of inflammatory cells infiltration, substantial interstitial cellular degeneration and necrosis, and exudate blockage or rupture of capillaries in the lung and liver. Then, the levels of serum inflammatory cytokines were detected. As shown in Fig. 1D to F, compared with *Hsf1^+/+^* endotoxemic mice, the levels of serum IL-6, IL-1β, and TNF-α were significantly higher in *Hsf1^−/−^* endotoxemic mice. These results suggested that HSF1 attenuated the release of inflammatory cytokines in endotoxemic mice. In order to examine whether the release of inflammatory cytokines in endotoxemic mice was associated with autophagy, autophagy markers LC3-II and LC3-I were examined by Western blot. As shown in Fig. 1G and H, the ratio of LC3-II/LC3-I decreased significantly in *Hsf1^−/−^* endotoxemic mice liver and lung compared with those in *Hsf1^+/+^* endotoxemic mice. These results suggest that HSF1 decreases the releasing of inflammatory cytokines and enhances LPS-induced autophagy in endotoxemic mice.

### mRNA levels of six autophagy-related genes were detected in *Hsf1^+/+^* and *Hsf1^−/−^* peritoneal macrophages.

**FIG 2:**
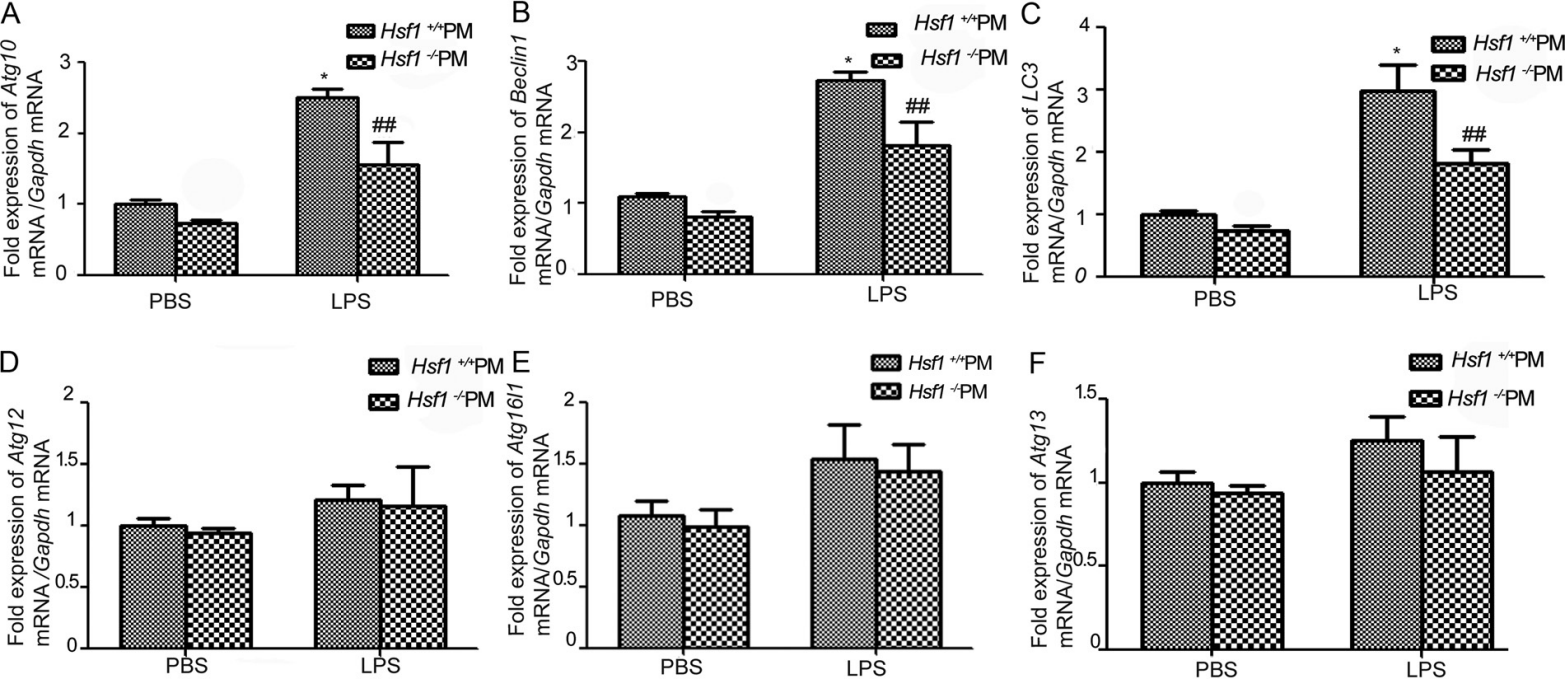
mRNA levels of six autophagy-related genes were detected in *Hsf1^+/+^* and *Hsf1^−/−^* peritoneal macrophages. (A to F) mRNA of autophagy-related genes *Atg10*, *LC3*, *Beclin1*, *Atg12*, *Atg16l1*, and *Atg13* was detected by RT-PCR. *, *P < *0.05 versus *Hsf1^+/+^* PM treated with PBS (*n* = 3); **^##^**, *P < *0.01 versus *Hsf1^+/+^* PM treated with LPS (*n* = 3).

Six autophagy-related genes *Atg10, LC3, Beclin1, Atg12, Atg16l1*, and *Atg13*, which promoter region contained HSF1 binding elements (nGAAnnTTCn or nTTCnnGAAn), were selected (Table 1). mRNA expression levels of these genes were detected by real-time PCR (RT-PCR). The results showed that the mRNA levels of *Atg10*, *LC3*, and *Beclin1* decreased significantly in *Hsf1^−/−^* peritoneal macrophages compared with those in *Hsf1^+/+^* peritoneal macrophages (Fig. 2A to C). However, there was no obvious difference in the expression levels of *Atg12*, *Atg16l1*, and *Atg13* between *Hsf1^+/+^* and *Hsf1^−/−^* peritoneal macrophages (Fig. 2D to F). These results indicated that *Atg10*, *LC3*, and *Beclin1* might be involved in *Hsf1*^+/+^ peritoneal macrophage autophagy induced by LPS.

**TABLE 1:** Autophagy-related genes which the promoter region containing HSE^*a*^

GenBank	Symbol	Description	HSE	HSE sequence	Position (transcription starting point + 1)
NM_026217	*ATG12*	Autophagy-related protein 12	2	ATTCAAGAAG	−1764 to −1755
				GTTCTAGAAGAGAACTTTCA	−1265 to −1246
NM_145528	*ATG13*	Autophagy-related protein 13	1	TTTCCCGAAT	−1646 to −1637
NM_025770	*ATG10*	Autophagy-related protein 10	1	AGAATATTCA	−1758 to −1749
NM_029846	*ATG16 L 1*	Autophagy-related protein 16-1	3	GGAACATTCC	−1950 to −1941
				GGAACTTTCC	−953 to −994
				TTTCTGGAAA	−784 to −775
NM_019584	*Beclin1*	Coiled-coil myosin-like BCL2-interacting protein	2	AGAAGATTCC	−1294 to −1285
				GGAAGTTTCC	−1398 to −1389
NM_025735	*LC3α*	Microtubule-associated protein 1 light chain 3 alpha	2	GTTCCAGAAT	−366 to −357
				CTTCTCGAAT	−142 to −133

*^a^*HSE, heat shock element (nGAAnnTTCn or nTTCnnGAAn).

### HSF1 can bind to promoter regions of *Atg10*.

**FIG 3:**
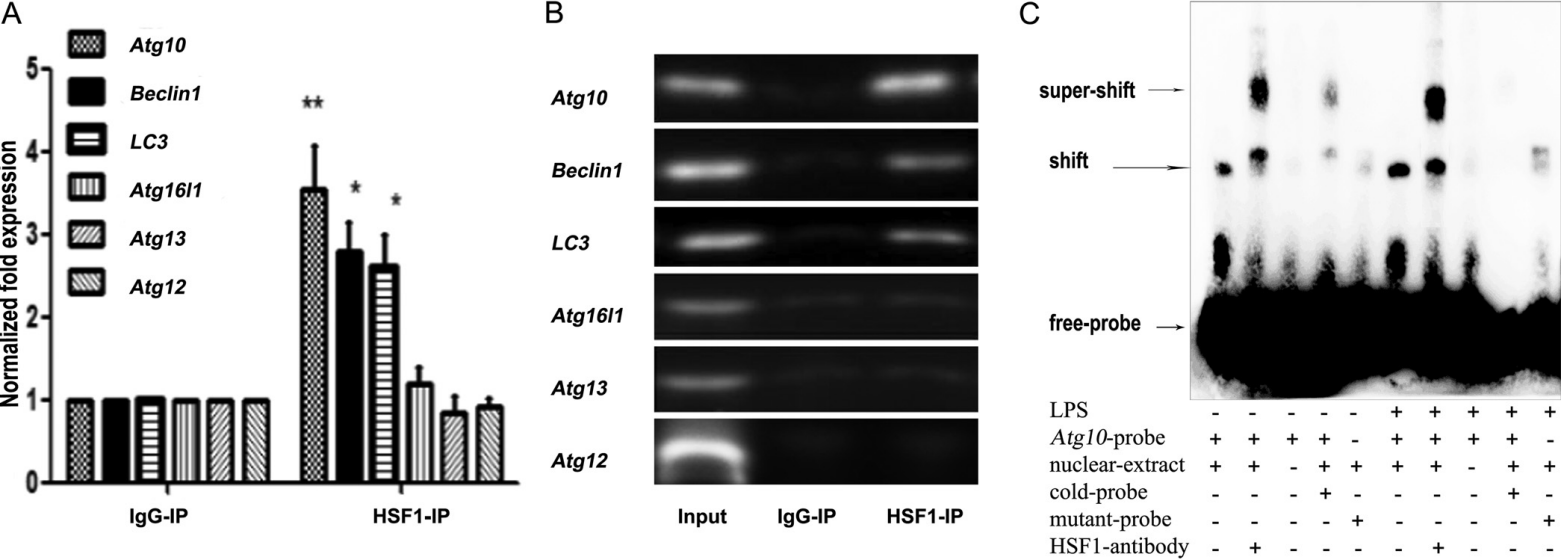
HSF1 binds to autophagy-related gene promoter region. (A and B) ChIP assays were performed using chromatin isolated from RAW264.7 cells. Antibody against HSF1 was used for immunoprecipitation (HSF1-IP). Antibody against IgG was used as a negative control (IgG-IP). The total DNA (input) was used as the positive control. Precipitates were quantified by qRT-PCR using specific primers of *Atg10*, *LC3*, *Beclin1*, *Atg12*, *Atg16l1*, and *Atg13*. *, *P < *0.05; **, *P < *0.01 versus IgG-IP (*n* = 3). (C) EMSA was performed with the nuclear extracts from untreated or treated with LPS RAW264.7 cells using an *Atg10* probe. The arrows indicated the specific binding bands (shift) and supershifted bands of higher molecular weight (supershift). Biotin-labeled free probe and mutation probe sequences were detailed in the Materials and Methods section. Cold probes were not labeled with biotin, more than 200 times as many as free probes. The experiment shown was representative of three similar experiments.

To explore the mechanism of HSF1 regulate autophagy, a chromatin immunoprecipitation (ChIP) assay and electrophoretic mobility shift assay (EMSA) were performed. Nuclear fractions were extracted from RAW264.7 cells, and the DNAs were immunoprecipitated by anti-HSF1 monoclonal antibody and analyzed by quantitative real-time PCR (qRT-PCR). As shown in Fig. 3A and B, *Atg10*, *LC3*, and *Beclin1* existed in the precipitate, and the quantity of *Atg10* was the highest. However, *Atg12*, *Atg16l1*, and *Atg13* were not found in the precipitate. EMSA was used to detect whether HSF1 could bind to the promoter region of autophagy-related genes *in vitro*. As shown in Fig. 3C, HSF1 could bind to the *Atg10* promoter region in RAW264.7 cells *in vitro*. The stronger binding band was observed between HSF1 and *Atg10* when treated with LPS (Fig. 3C, lane 6 versus lane 1). Supershifted bands of a higher molecular weight were observed when an anti-HSF1 antibody was added (Fig. 3C, lane 2 and lane 7). The binding reaction did not occur when the cold and mutant probe were added (Fig. 3C, lanes 4 and 5 versus lanes 9 and 10). These results confirmed that HSF1 specifically bound the noncoding region of the *Atg10* promoter *in vitro*. However, no obvious binding band or supershifted band was traced between HSF1 and *LC3*, *Beclin1*, *Atg12*, *Atg16l1*, and *Atg13* (data not shown).

### HSF1 upregulated the protein expression of ATG10 and increased the ratio of LC3-II/LC3-I in peritoneal macrophages and RAW264.7 cells.

**FIG 4:**
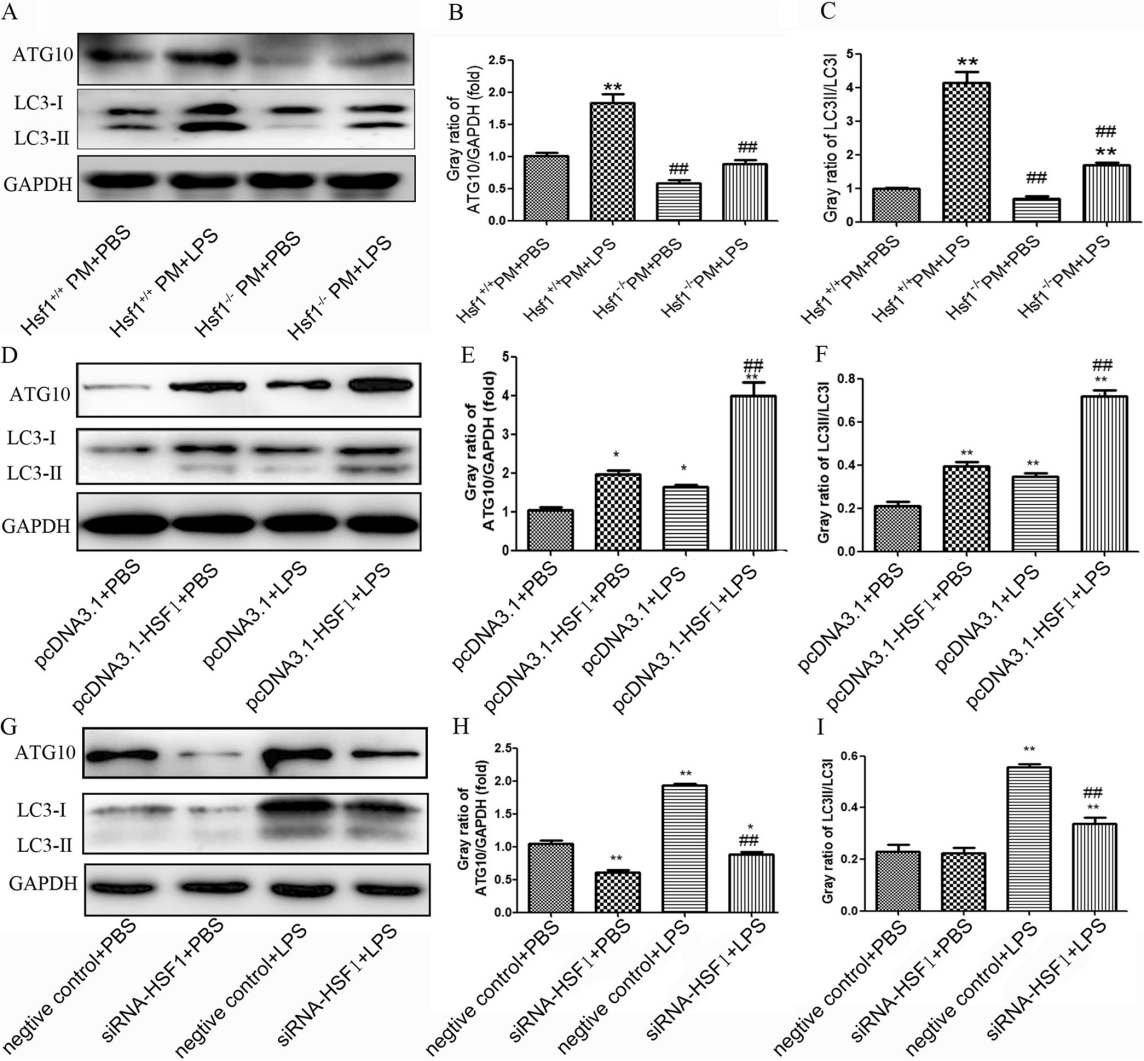
HSF1 upregulated the expression of the ATG10 protein and increased the ratio of LC3-II/LC3-I in peritoneal macrophages and RAW264.7 cells. (A to C) Primary peritoneal macrophages (PM) were obtained from *Hsf1*^−/−^ and *Hsf1^+/+^* mice and treated with 1,000 ng/mL LPS for 8 h. The expression of ATG10, LC3-II, and LC3-I proteins were measured with anti-ATG10 and anti-LC3 antibody in *Hsf1*^−/−^ and *Hsf1^+^*^/+^PM; **, *P < *0.01 versus *Hsf1^+/+^*PM +PBS (*n* = 3); ^##^, *P < *0.01 versus *Hsf1^+/+^*PM +LPS (*n* = 3). (D to F) RAW264.7 cells were transfected with pcDNA3.1-HSF1 or pcDNA 3.1 for 48 h and treated with LPS (1,000 ng/mL) for another 8 h. The protein levels of ATG10 and LC3 were tested by Western blotting. *, *P < *0.05; **, *P < *0.01 versus pcDNA3.1+PBS (*n* = 3); **^##^**, *P < *0.01 versus pcDNA3.1+LPS (*n* = 3). (G to I) RAW264.7 cells were transfected with siRNA-HSF1 or negative control for 48 h and treated with LPS (1,000 ng/mL) for another 8 h. Then the protein levels of ATG10 and LC3 were tested by Western blotting. *, *P < *0.05; **, *P < *0.01 versus negative control+PBS (*n* = 3); **^##^**, *P < *0.01 versus negative control+LPS (*n* = 3).

The above results suggested that HSF1 might upregulate autophagy through transcriptional regulation of the expression of *Atg10*. Therefore, we selected *Atg10* for further study. First, primary peritoneal macrophages (PM) were obtained from *Hsf1*^−/−^ and *Hsf1^+/+^* mice and treated with 1,000 ng/mL of LPS for 8 h. As shown in Fig. 4A to C, compared with *Hsf1*^+/+^ PM+phosphate-buffered saline (PBS), the expression of ATG10 and the ratio of LC3-II/LC3-I were increased in *Hsf1* ^+/+^ PM+LPS. However, the expression of ATG10 and the ratio of LC3-II/LC3-I were obviously decreased in *Hsf1*^−/−^ PM+LPS versus *Hsf1*^+/+^ PM+LPS. Overexpression of HSF1 increased the expression level of ATG10 and enhanced the ratio of LC3-II/LC3-I in RAW264.7 cells treated with LPS (Fig. 4D to F). In contrast, silencing of HSF1 decreased the expression of ATG10 and markedly lowered the ratio of LC3-II/LC3-I in RAW264.7 cells treated with LPS (Fig. 4G to I). These results indicated that HSF1 enhanced LPS-induced autophagy through transcriptional regulation of the expression of ATG10 in PM and RAW264.7 cells.

### siRNA-ATG10 suppressed HSF1-mediated autophagy.

**FIG 5:**
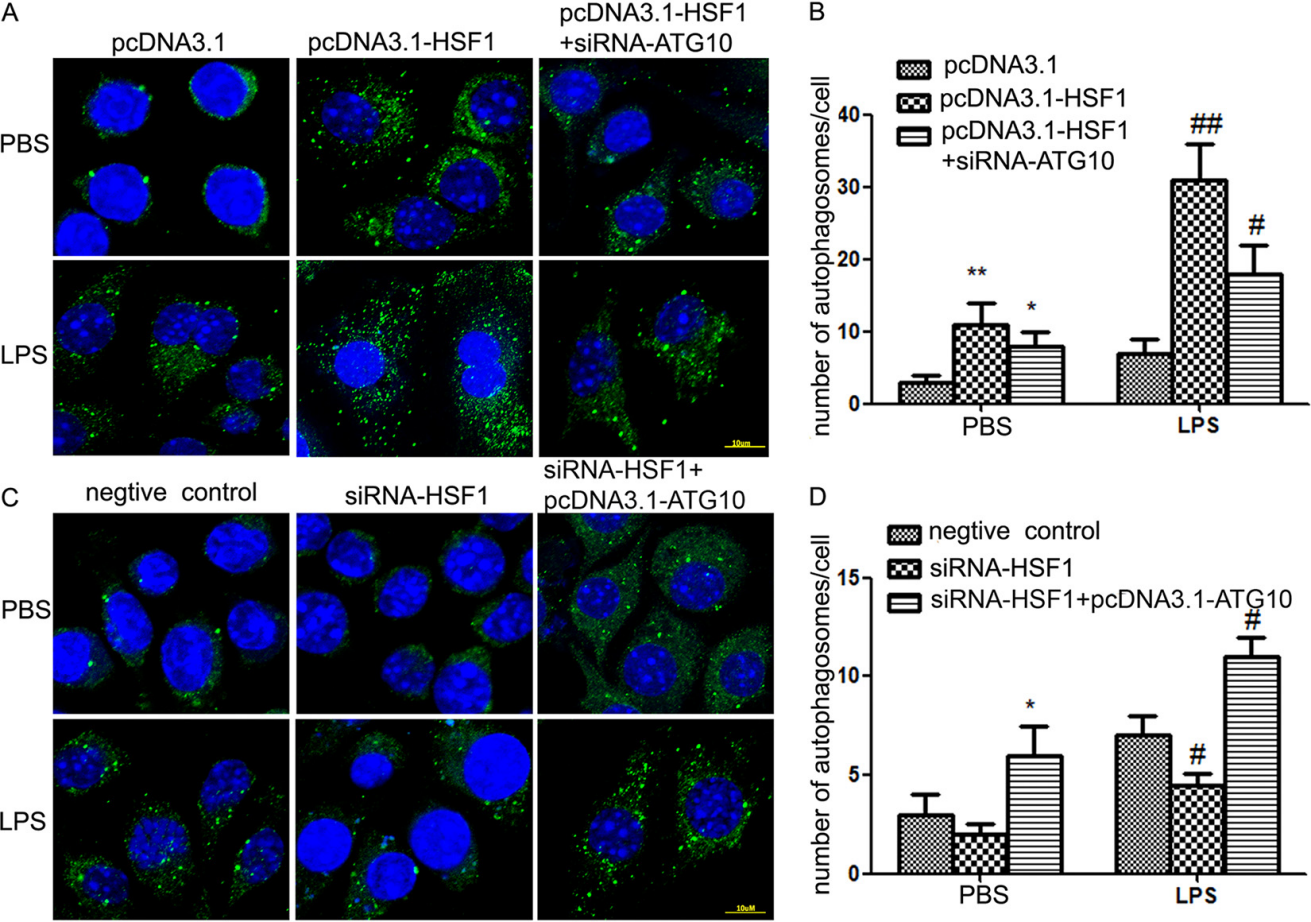
siRNA-ATG10 abrogates HSF1-mediated autophagy. LPS-induced autophagy was measured by immunofluorescence detection. The cells were incubated with anti-LC3 primary antibodies. The secondary fluorescence antibodies were goat anti-rabbit Alexafluor 488 (green fluorescence). Nuclei were stained with DAPI (blue fluorescence). (A and B) RAW264.7 cells were transfected with pcDNA3.1-HSF1 or pcDNA3.1-HSF1+siRNA-ATG10. Scale bar, 10 μm. Left, shows were representative photos; right, shows quantitative analysis; at least 30 cells were calculated. *, *P < *0.05; **, *P < *0.01 versus pcDNA3.1+PBS; *n* ≥ 5. ^#^, *P < *0.05; ^##^, *P < *0.01 versus pcDNA3.1+LPS; *n* ≥ 5. (C and D) RAW264.7 cells were transfected with siRNA-HSF1 or siRNA-HSF1+pcDNA3.1-ATG10; scale bar, 10 μm. Left, shows representative photos; right, shows quantitative analysis; at least 30 cells were calculated. *, *P < *0.05 versus negative control + PBS; *n* ≥ 5. ^#^, *P < *0.05 versus negative control + LPS; *n* ≥ 5.

To investigate the role of *Atg10* in the process of HSF1-mediated autophagy, immunofluorescence assays of LC3 were performed in RAW264.7 cells. PcDNA3.1-HSF1 was transfected in RAW264.7 cells. The results found that overexpression of HSF1 increased the numbers of autophagosomes/cell. In the rescue experiment, when pcDNA3.1-HSF1 and siRNA-ATG10 were cotransfected, decreased numbers of autophagosomes/cell were detected compared with only pcDNA3.1-HSF1-transfected RAW264.7 cells (Fig. 5A and B). When cotransfected siRNA-HSF1 with pcDNA3.1-ATG10, increased numbers of autophagosomes/cell were detected compared with only siRNA-HSF1-transfected RAW264.7 cells (Fig. 5C and D).

### siRNA-ATG10 negated HSF1-mediated attenuation of the inflammatory cytokine release.

**FIG 6:**
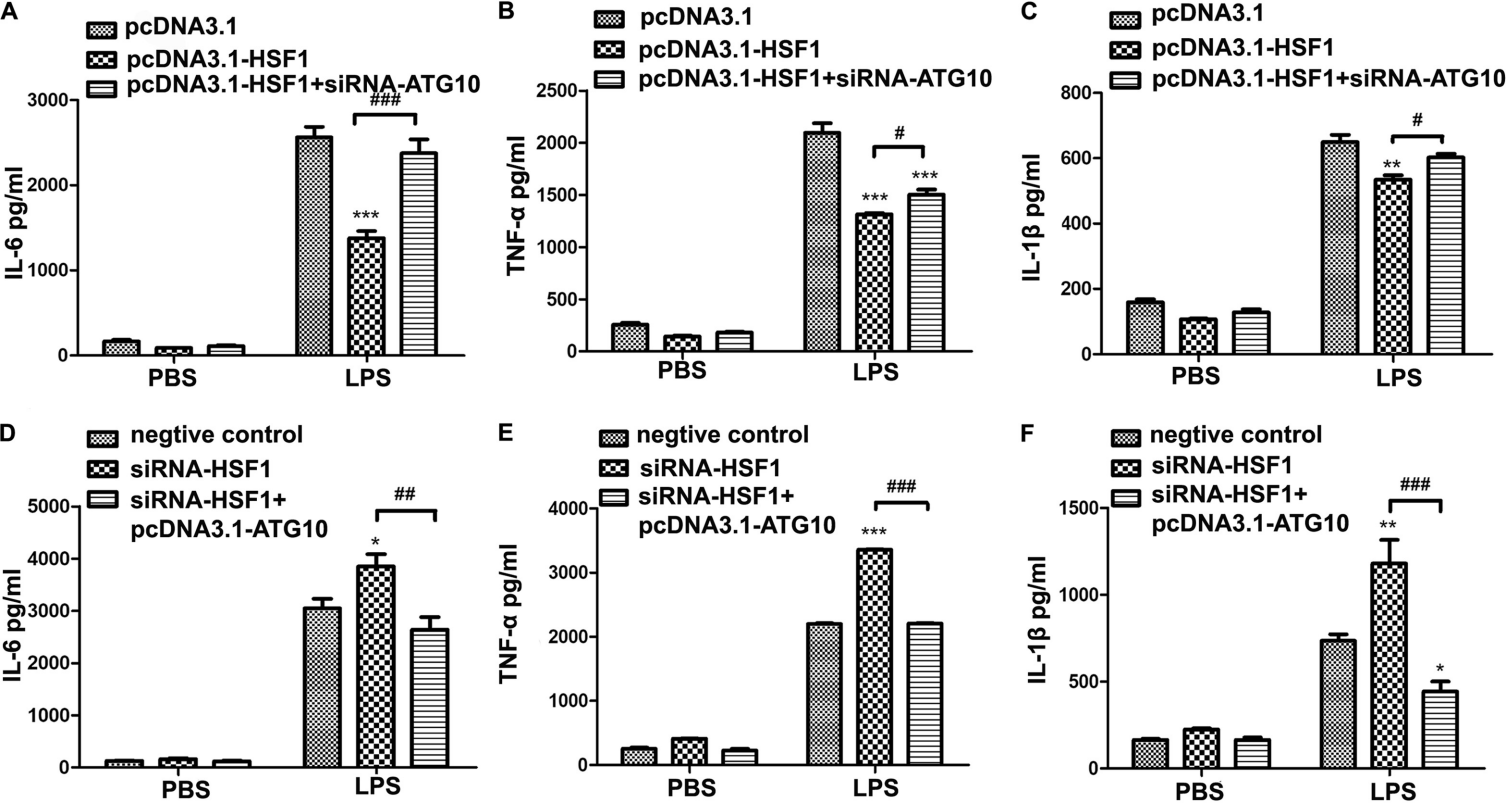
siRNA-ATG10 abrogated HSF1-mediated attenuation of the release of inflammatory cytokines. (A to C) LPS-induced IL-6, TNF-α, and IL-1β in the culture medium were measured by ELISA. **, *P < *0.01 and ***, *P < *0.001 versus pcDNA3.1+LPS, *n* ≥ 5; **^#^**, *P < *0.05 and **^###^**, *P < *0.001 versus pcDNA3.1-HSF1+LPS, *n* ≥ 5. (D to F). LPS-induced IL-6, TNF-α, and IL-1β in the culture medium were measured by ELISA. *, *P < *0.05; **, *P < *0.01; ***, *P < *0.001 versus negative control+LPS; *n* ≥ 5. **^##^**, *P < *0.01 and **^###^**, *P < *0.001 versus siRNA-HSF1+LPS; *n* ≥ 5.

In order to determine whether HSF1-mediated attenuation of the release of inflammatory cytokines is related to *Atg10*, inflammatory cytokines were detected in RAW264.7 cells. As shown in Fig. 6A to C, compared with the HSF1 overexpression in RAW264.7 cells, the release of the cytokines IL-6, IL-1β, and TNF-α were significantly increased (*P < *0.01, *n* ≥ 5) in pcDNA3.1-HSF1 with siRNA-ATG10-cotransfected RAW264.7 cells treated with LPS. In contrast, compared with the siRNA-HSF1 RAW264.7 cells, the release of IL-6, IL-1β, and TNF-α were significantly decreased (*P < *0.01, *n* ≥ 5) in siRNA-HSF1 and pcDNA3.1-ATG10 cotransfected RAW264.7 cells (Fig. 6D to F). Thus, HSF1-induced protection from endotoxemia was largely *Atg10* dependent.

## DISCUSSION

HSF1 is a transcription factor that regulates eukaryotic heat shock proteins (HSPs), which are highly conserved among different species (23). HSF1 also regulates non-heat shock genes, including autophagy-related genes. For instance, HSF1 plays a key role in chemotherapeutic agent-induced cytoprotective autophagy through the transcriptional upregulation of autophagy-related gene 7 (Atg7) (24). Another report has shown that HSF1 upregulates the expression of ATG4B in hepatoma carcinoma cells by binding to the promoter region of the gene and consequently enhancing the epirubicin (EPI)-induced protective autophagy, which in turn promotes hepatocellular carcinoma (HCC) cell survival (25). Samarasinghe et al. (26) have found that sequestosome 1 (p62/SQSTM1), a protein involved in the delivery of autophagic substrates and nucleation of autophagosomes, is an HSF1-regulated gene. Du et al. (27) found that silencing HSF1 correlated with a reduction in autophagy and thus enhanced cell death. Compared with the wild-type subgroup, the expressions of LC3II/LC3I and Beclin1 were shown to have an obvious increase, and increased autophagosome formation was observed on electron microscopy in the HSF1 transgene (HSF1-TG) subgroup. Opposite results were observed in the HSF1-KO subgroup. These results suggest that HSF1 promoted autophagosome formation.

Based on the above results, we speculated that HSF1 could participate in autophagy by transcriptionally regulating the expression of autophagy-related genes. Therefore, six autophagy-related genes (*Atg10*, *LC3*, *Beclin1*, *Atg12*, *Atg16l1*, and *Atg13*) were selected, which promoter region-containing HSF1 binding elements (nGAAnnTTCn or nTTCnnGAAn). The results showed that the mRNA levels of *Atg10*, *LC3*, and *Beclin1* were increased significantly in *Hsf1^+/+^* peritoneal macrophages compared with those in *Hsf1^−/−^* peritoneal macrophages by RT-PCR (Fig. 2). So, we speculated that *Atg10*, *LC3*, and *Beclin1* may be the transcriptional target genes of HSF1. Then, a ChIP assay was used to detect the interaction relationship between HSF1 and these genes. The ChIP assay results found that *Atg10*, *LC3*, and *Beclin1* were in the precipitate and that the quantity of *Atg10* was the highest. It indicated that *Atg10* was mainly a transcriptional target gene of the HSF1 (Fig. 3). EMSAs were employed to investigate the transcriptional target of HSF1. In the experiment of EMSA, a shift band and supershifted band were observed in *Atg10* and HSF1. However, only a shift band was observed in *LC3* or *Beclin1* and HSF1, and no supershifted band of higher molecular weight was observed in *LC3* or *Beclin1*. We considered that there may be two reasons for this finding, as follows: first, an antibody to HSF1 blocks the binding site, and second, there is no specific binding between HSF1 and *LC3* or *Beclin1*. These ideas remain to be further studied.

Based on the above finding, we selected *Atg10* for further study. Further studies found that the overexpression of HSF1 increased the expression level of *Atg10* and enhanced the induction of autophagy in RAW264.7 cells. In turn, silencing of HSF1 inhibited the expression of *Atg10* and autophagy (Fig. 4).

*Atg10* is necessary for the occurrence of autophagy, it is a ubiquitin-binding (E2) enzyme mimic that conjugates *Atg12* to *Atg5*, and it is essential for yeast autophagy (28, 29). There is also evidence that *Atg10* has a significant effect on autophagy. In Crassostrea gigas, the mRNA expression of *Atg10* in the mantle of oysters was significantly upregulated after poly (I·C) stimulation. The formation of autophagosome expression was inhibited in *Atg10*-knockdown oysters. *Atg10* was involved in the formation of autophagosome and antivirus immune response of oysters (30). In the substantia nigra of a Parkinson’s disease (PD) rat model, *p62*, *Atg5*, *Atg12*, *LC3*, and *Atg16l1* were expressed, while *Atg10* was not expressed. This result indicates that *Atg10* expression is necessary for the initiation of autophagy in the rat model of PD (31). ATG10 also plays an important role other than autophagy in diseases. Jo et al. (32) reported that the expression of ATG10 is associated with lymph node and lymphatic vessel metastasis in colorectal cancer. Moreover, ATG10 modulated epithelial mesenchymal-associated protein depletion in colorectal cancer cells (33). The c-Myc/miR-27b-3p/ATG10 signaling pathway was involved in regulating colorectal cancer chemoresistance (34). However, the role of ATG10 in endotoxemia has not been studied.

In order to determine whether HSF1-mediated protective autophagy and protective effect are related to *Atg10* in endotoxemia, cotransfection of pcDNA3.1-HSF1 with siRNA-ATG10 or siRNA-HSF1 with pcDNA3.1-ATG10 was performed in RAW264.7 cells. Compared with the HSF1 overexpression group, the numbers of autophagosomes per cell was clearly lower in pcDNA3.1-HSF1 with siRNA-ATG10-cotransfected RAW264.7 cells (Fig. 5A and B). Compared with that of the siRNA-HSF1-transfected cells, the numbers of autophagosomes per cell were clearly increased in siRNA-HSF1 with pcDNA3.1-ATG10-cotransfected RAW264.7 cells (Fig. 5C and D). Similar trends are observable in the release of the cytokines IL-6, IL-1β, and TNF-α (Fig. 6).

In this study, we provided multiple lines of evidence that HSF1 attenuated the release of inflammatory cytokines in endotoxemia mice by upregulating *Atg10*. First, we found that the levels of *Atg10* mRNA were elevated in LPS-treated *Hsf1^+/+^* peritoneal macrophages. Second, HSF1 can directly bind to the promoter region of *Atg10* in RAW264.7 cells. Third, overexpression of HSF1 increased the expression of ATG10 and enhanced the ratio of LC3-II/LC3-I in RAW264.7 cells. In contrast, silencing of HSF1 decreased the expression of ATG10 and markedly lowered the ratio of LC3-II/LC3-I. Finally, silencing *Atg10* with siRNA negated the protective effect of HSF1 in RAW264.7 cells treated with LPS.

Thus, HSF1-induced protection from endotoxemia was largely *Atg10* dependent. However, the role of *Atg10* in endotoxemia other than autophagy and the transcriptome change in HSF1-deficient cells have not been studied, which will be our interesting research avenue in the future.

## MATERIALS AND METHODS

### Mouse model of endotoxemia.

*Hsf1* knockout (*Hsf1^−/−^*) and wild-type (*Hsf1*^+/+^) mice were provided by Ivor J. Benjamin (University of Utah, Salt Lake City, UT) and had been described elsewhere (35). Endotoxemia was induced in mice as described previously (8). Mice homozygous for the *Hsf1* knockout allele were maintained along with age-matched control littermates. *Hsf1^−/−^* and *Hsf1^+/+^* mice (*n* = 6 to 8 per group) were injected intraperitoneally with 10 mg/kg of body weight of LPS (Escherichia coli 0111: B4; Sigma-Aldrich) diluted in pyrogen-free normal saline (NS). *Hsf1^−/−^* and *Hsf1^+/+^* mice (*n* = 6 to 8 per group) injected with NS were used as the sham. Lungs, livers, and sera were harvested 12 h after injections. Then the mice were euthanized. The investigation followed the Guide for the Animal Care and Use Committee of Central South University. The study protocol was approved by the Ethics Committee of Central South University, Medical Institution Animal Care and Research Advisory Committee (Changsha, China).

### Isolation of peritoneal macrophages and RAW264.7 cell culture.

Peritoneal macrophages (PM) were collected from *Hsf1^+/+^* and *Hsf1^−/−^* mice by peritoneal lavage with Dulbecco’s phosphate-buffered saline as described previously (8). Thioglycolate-elicited peritoneal cells were obtained from animals 3 days after intraperitoneal injection of 3 mL thioglycolate broth (Sigma-Aldrich, St. Louis, MO). Macrophages were further purified by adherence to culture dishes for 24 h and identified using anti-HSF1 antibodies (Santa Cruz Biotechnology, Santa Cruz, CA) before use. After 24 h of *in vitro* culture, peritoneal macrophages were treated with 1,000 ng/mL of LPS for 8 h. RAW264.7 cells (mouse leukemia cells of monocyte macrophage) were purchased from the Shanghai Cell Bank of the Chinese Academy of Sciences (Shanghai, China). The cells were cultured with Dulbecco’s modified Eagle medium (DMEM) plus 10% fetal bovine serum under 5% CO_2_ conditions at 37°C. RAW264.7 cells were treated with 1,000 ng/mL of LPS for 8 h.

### Histopathological examination.

The livers and lungs were fixed in 10% neutral buffered formalin at room temperature for 24 h. The samples were dehydrated in ethanol of increasing concentration gradient (75%, 85%, 95%, and 100%), cleared in xylene for 2 h, soaked in paraffin for 2 h, and then embedded. Sections were cut at 4-μm thickness and placed on glass slides. To deparaffinize samples, the slides were placed in xylene and then in ethanol of a decreasing concentration gradient. Slides were stained with hematoxylin for 5 min and then with eosin for 10 s, and finally they were dehydrated by a gradient of alcohol and xylene. Then the samples were sealed with neutral balsam on slides and examined by an experienced pathologist under a light microscope (Nikon, Tokyo, Japan). Semiquantitative analysis about the histopathological injury of livers was evaluated as described previously by Mohamed et al. (36). Briefly, liver injury was graded from 0 (normal) to 4 (severe) in four categories, as follows: hepatocellular necrosis, hemorrhage, hepatic parenchymal inflammatory infiltrate, and sinusoidal inflammatory infiltrate. Semiquantitative analysis about the histopathological injury of lungs was evaluated as described previously by Yousef et al. (37). Briefly, lung injuries were defined as neutrophil infiltration, interstitial edema, congestion, hemorrhage, hyaline membrane formation, and necrosis. The severity of microscopic injury was judged according to the following scoring system: 0, normal; 1, minimal (<25%); 2, mild (25% to 50%); 3, moderate (50% to 75%); and 4, severe (>75%). Tissue sections were examined in by an experienced pathologist who was blind to treatments.

### Total RNA extraction and reverse transcription-PCR analysis.

Total RNA was isolated from RAW264.7 cells using TRIzol reagent (Thermo Fisher, USA). Total RNA was used to reverse transcribe a cDNA template using a PrimeScript RT reagent kit (RR037A; TaKaRa, Dalian, China). PCRs were performed using SYBR Premix *Ex Taq*II (RR820A; TaKaRa, Dalian, China). The PCR assays were conducted according to the manufacturer’s instructions. The sequences are listed for each of the following genes: *Atg10* left primer, 5′-GGAGAACAGCCAAGGAAT-3′; *Atg10* right primer, 5′-CTCGTCACTTCAGAATCATC-3′; *Beclin1* left primer, 5′-TGGATGACGAACTCAAGAG-3′; *Beclin1* right primer, 5′-GATGTGGAAGGTGGCATT-3′; *LC3A* left primer, 5′-GAGCGAGTTGGTCAAGAT-3′; *LC3A* right primer, 5′-TCATAGATGTCAGCGATGG-3′; *Atg12* left primer, 5′-ATCCTGCTGAAGGCTGTA-3′; *Atg12* right primer, 5′-TGATGAAGTCAATGAGTCCT-3′; *Atg13* left primer, 5′-GGAGATTCTATGGAGTTGGA-3′, *Atg13* right primer, 5′-TTCAGCAGCAGTGACAAT-3′; *Atg16l1* left primer, 5′-CGCTCTGTCTCTTCCATC-3′; and *Atg16l1* right primer, 5′-ACATACGAGGCAGTAGTTG-3′.

### Immunofluorescence analysis.

First, RAW264.7 cells were seeded on glass covers lips in a 24-well culture plate. Second, cells were fixed in 4% paraformaldehyde for 10 min and washed three times with cold PBS. Then, cells were incubated with the primary antibody LC3 (MBL) at a dilution of 1:1,000 for 1 h after blocking with 10% fetal bovine serum or 5% bovine serum albumin (in PBS) for 30 min. Last, the cells were incubated with a second fluorescence antibody, Alexafluor 488 goat anti-rabbit antibody (Invitrogen, Carlsbad, A), at a dilution of 1:1,000. Nuclei were stained with 4′,6-diamidino-2-phenylindole (DAPI; C1006; Beyotime Biotechnology) for 5 min at room temperature. Cells were imaged with a confocal microscope (Leica Camera AG, Oskar-Barnack-StraQe, Germany) after the glass coverslip was mounted with glycerol and at least 30 cells were counted.

### Enzyme-linked immunosorbent assay (ELISA).

Cytokines (IL-6, IL-1β, and TNF-α) in the serum and cell culture medium were measured in accordance with the manufacturer’s instructions from commercially available enzyme-linked immunosorbent assay kits (Boster Biological Technology, Wuhan, Hubei, China). The experiments were repeated at least five times, and the data shown are the means ± SD.

### Protein preparation and Western blot.

Proteins from the liver and lung tissues, peritoneal macrophages, and RAW264.7 cells were extracted in radioimmunoprecipitation assay (RIPA) buffer (1% Triton X-100, 10 mM Tris-HCl, 5 mM EDTA, and 150 mM NaCl [pH 7.0]) containing a protease inhibitor cocktail. Protein extracts were subjected to centrifugation at 12,000 *g* for 20 min. The protein concentration was determined using a bicinchoninic acid (BCA) protein assay kit (Dingguo Changsheng Biotechnology, Beijing, China). Total protein was separated on a 12% or 15% sodium dodecyl sulfate-polyacrylamide gel and transferred to an polyvinylidene fluoride (FVDF) membrane (Millipore, Billerica, MA). The membranes were then incubated overnight at 4°C with a primary antibody against LC3 (MBL, Japan) at a dilution of 1:1,000, GAPDH (Sigma-Aldrich) at a dilution of 1:1,000, HSF1 (Santa Cruz Biotechnology) at a dilution of 1:500, ATG10 (Cell Signaling Technology) at a dilution of 1:1000, or β-actin (Sigma-Aldrich) at a dilution of 1:1,000. Subsequently, the membranes were washed three times with Tris-buffered saline with Tween 20 (TBST; pH 7.6) and incubated with horseradish peroxidase-conjugated goat or rabbit IgG antibody (diluted 1:2,000 in TBST buffer) for 1 h at room temperature. The membranes were then washed with TBST three times. The membranes were visualized by an ECL detection kit (Thermo Fisher, USA), and the experiments were repeated at least three times.

### Plasmid constructs.

The plasmid construct pcDNA3.1-ATG10 was created as follows. *Atg10* (GenBank NM_025770.3) was amplified by PCR with Pyrobest (TaKaRa, Dalian, China). The PCR products were cloned into the pcDNA3.1 vector at cloning sites NheI-BamHI. The putative pcDNA3.1-ATG10 was verified by DNA sequencing (Sangon Biotech, Shanghai, China). pcDNA3.1-HSF1 was constructed by other members of our research group and has been described elsewhere (38).

### Transfect experiments.

After the cells reached approximately 80% confluence, RAW264.7 cells were transfected with pcDNA3.1-HSF1 plasmid or pcDNA3.1-ATG10 plasmid according to the manufacturer’s instructions (Lipofectamine 2000; Invitrogen, Carlsbad, CA). siRNA-HSF1 (Invitrogen, Carlsbad, CA) and siRNA-ATG10 (Invitrogen, Carlsbad, CA) were transfected by the HiPerFect transfection reagent according to the manufacturer’s instructions (Qiagen, Cambridge, MA).

### Chromatin immunoprecipitation (ChIP) assay.

The ChIP assay was performed using a ChIP assay kit (RK20100; Abclonal, Wuhan, China) according to the instructions of the manufacturer. RAW264.7 cells were fixed with 1% formaldehyde for 10 min and lysed with lysis buffer. Then the chromatin fragmentation was performed by using a microtip probe sonicator to shear DNA and was immunoprecipitated with an HSF1 antibody (Santa Cruz Biotechnology); rabbit IgG was used as the negative control. The DNA amounts of *Atg10*, *LC3A*, *Beclin1*, *Atg12*, *Atg13*, and *Atg16l1* in the precipitate were detected by quantitative real-time PCR (qRT-PCR). The primer sequences of the following At*g10*, *LC3A*, *Beclin1*, *Atg12*, *Atg13*, and *Atg16l1* promoter regions are listed: *Atg10* left primer, TCTTGGCATTCTTCATGCTG; *Atg10* right primer, GTTCCAACAGCGACCTATCC; *Beclin1* left primer, CAGCTGCAGGGGTAAGAGAC; *Beclin1* right primer, CCTGTGCTGGTTTCCAATTT; *LC3A* left primer, TTGTTCCTCTGTGGCCTCTT; *LC3A* right primer, AGAGTACATGCCTCCCATGC; *Atg12* left primer, ATCCTGCTGAAGGCTGTA; *Atg12* right primer, TGATGAAGTCAATGAGTCCT; *Atg13* left primer, GGAGATTCTATGGAGTTGGA; *Atg13* right primer, TTCAGCAGCAGTGACAAT; *Atg16l1* left primer, CGCTCTGTCTCTTCCATC; and *Atg16l1* right primer, ACATACGAGGCAGTAGTTG.

### Electrophoretic mobility shift assay (EMSA).

Nuclear and cytosolic proteins were prepared from cells with NE-PER nuclear and cytoplasmic extraction reagents (Pierce Biotechnology, Rockford, USA). Biotin-labeling oligonucleotides for autophagy-related genes were synthesized by SongGon Biotech (Wuhan, China). The electrophoretic mobility shift assay (EMSA) reactions were prepared according to the manufacturer’s protocol (Light Shift Chemiluminescent EMSA kit; Thermo Fisher Scientific, Waltham, MA). The following are the probe, cold probe, and mutant probes of autophagy-related genes and their sequences: *Atg10* probe, TGGGGGCTGAGGCAGAATATTCAAATATGACGG; *Atg10* cold probe, TGGGGGCTGAGGCAGAATATTCAAATATGACGG; *Atg10* mutant probe, TGGGGGCTGAGGCACAATAGGCAAATATGACGG; *Beclin1* probe, TTGGAAGCAATGGAAGTTTCCATCAATAAAGAA; *Beclin1*cold probe, TTGGAAGCAATGGAAGTTTCCATCAATAAAGAA; *Beclin1* mutant probe, TTGGAAGCAATGGCCGTTTGGATCAATAAAGAA; *LC3A* probe, CTTTCTATGGTTGTTCCAGAATTCTTTGAAAGCCTG; *LC3A* cold probe, CTTTCTATGGTTGTTCCAGAATTCTTTGAAAGCCTG; *LC3A* mutant probe, CTTTCTATGGTTGGGCCACAATTCTTTGAAAGCCTG; *Atg12* probe, AAACAATGTTCACAGTTCTAGAAGAGAACTTTC; *Atg12* cold probe, AAACAATGTTCACAGTTCTAGAAGAGAACTTTC; *Atg12* mutant probe, AAACAATGTTCACAGGGCTAGCCGAGAACTTC; *Atg13* probe, ATTTCTGGAAATAAGCCGCCTTTCTTGAAGAC; *Atg13* cold probe, ATTTCTGGAAATAAGCCGCCTTTCTTGAAGAC; *Atg13* mutant probe, ATGGCTGCAAATAAGCCGCCTGGCTTCAAGAC; *Atg16l1*probe, CCTGTGGGCTTTTCTGGAAAACCAGAAGTACC; *Atg16l1*cold probe, CCTGTGGGCTTTTCTGGAAAACCAGAAGTACC; and *Atg16l1* mutant probe, CCTGTGGGCTTGGCTGCAAAACCAGAAGTACC.

### Statistical analysis.

All quantitative data are presented as means ± SD. The differences between different groups were analyzed with one-way analysis of variance, followed by Student-Newman-Keuls or Dunnett’s test for *post hoc* comparisons. A *P* value of ≤ 0.05 was considered statistically significant.

## References

[1] Evans L, Rhodes A, Alhazzani W, Antonelli M, Coopersmith CM, French C, Machado FR, Mcintyre L, Ostermann M, Prescott HC, Schorr C, Simpson S, Wiersinga WJ, Alshamsi F, Angus DC, Arabi Y, Azevedo L, Beale R, Beilman G, Belley-Cote E, Burry L, Cecconi M, Centofanti J, Coz Yataco A, De Waele J, Dellinger RP, Doi K, Du B, Estenssoro E, Ferrer R, Gomersall C, Hodgson C, Møller MH, Iwashyna T, Jacob S, Kleinpell R, Klompas M, Koh Y, Kumar A, Kwizera A, Lobo S, Masur H, McGloughlin S, Mehta S, Mehta Y, Mer M, Nunnally M, Oczkowski S, Osborn T, Papathanassoglou E, et al. 2021. Surviving sepsis campaign: international guidelines for management of sepsis and septic shock 2021. Intensive Care Med 47:1181–1247. doi:10.1007/s00134-021-06506-y. 34599691 PMC8486643

[2] Basauri A, González-Fernández C, Fallanza M, Bringas E, Fernandez-Lopez R, Giner L, Moncalián G, de la Cruz F, Ortiz I. 2020. Biochemical interactions between LPS and LPS-binding molecules. Crit Rev Biotechnol 40:292–305. doi:10.1080/07388551.2019.1709797. 31931630

[3] Czamara K, Stojak M, Pacia MZ, Zieba A, Baranska M, Chlopicki S, Kaczor A. 2021. Lipid droplets formation represents an integral component of endothelial inflammation induced by LPS. Cells 10:1403. doi:10.3390/cells10061403. 34204022 PMC8227392

[4] Wang S, Tan KS, Beng H, Liu F, Huang J, Kuai Y, Zhang R, Tan W. 2021. Protective effect of isosteviol sodium against LPS-induced multiple organ injury by regulating of glycerophospholipid metabolism and reducing macrophage-driven inflammation. Pharmacol Res 172:105781. doi:10.1016/j.phrs.2021.105781. 34302975

[5] Mohammad S, Thiemermann C. 2020. Role of metabolic endotoxemia in systemic inflammation and potential interventions. Front Immunol 11:594150. doi:10.3389/fimmu.2020.594150. 33505393 PMC7829348

[6] Gauer R, Forbes D, Boyer N. 2020. Sepsis: diagnosis and management. Am Fam Physician 101:409–418. 32227831

[7] Molano FD, Arevalo-Rodriguez I, Roqué IFM, Montero Oleas NG, Nuvials X, Zamora J. 2019. Plasma interleukin-6 concentration for the diagnosis of sepsis in critically ill adults. Cochrane Database Syst Rev 4:CD011811. doi:10.1002/14651858.CD011811.pub2. 31038735 PMC6490303

[8] Tong Z, Jiang B, Zhang L, Liu Y, Gao M, Jiang Y, Li Y, Lu Q, Yao Y, Xiao X. 2014. HSF-1 is involved in attenuating the release of inflammatory cytokines induced by LPS through regulating autophagy. Shock 41:449–453. doi:10.1097/SHK.0000000000000118. 24430550

[9] Choudhury A, Bullock D, Lim A, Argemi J, Orning P, Lien E, Bataller R, Mandrekar P. 2020. Inhibition of HSP90 and activation of HSF1 diminish macrophage NLRP3 inflammasome activity in alcohol-associated liver injury. Alcohol Clin Exp Res 44:1300–1311. doi:10.1111/acer.14338. 32282939 PMC7328660

[10] Singh IS, He JR, Calderwood S, Hasday JD. 2002. A high affinity HSF-1 binding site in the 5'-untranslated region of the murine tumor necrosis factor-alpha gene is a transcriptional repressor. J Biol Chem 277:4981–4988. doi:10.1074/jbc.M108154200. 11734555

[11] Xie Y, Chen C, Stevenson MA, Auron PE, Calderwood SK. 2002. Heat shock factor 1 represses transcription of the IL-1beta gene through physical interaction with the nuclear factor of interleukin 6. J Biol Chem 277:11802–11810. doi:10.1074/jbc.M109296200. 11801594

[12] Takii R, Inouye S, Fujimoto M, Nakamura T, Shinkawa T, Prakasam R, Tan K, Hayashida N, Ichikawa H, Hai T, Nakai A. 2010. Heat shock transcription factor 1 inhibits expression of IL-6 through activating transcription factor 3. J Immunol 184:1041–1048. doi:10.4049/jimmunol.0902579. 20018623

[13] Gilchrist M, Thorsson V, Li B, Rust AG, Korb M, Roach JC, Kennedy K, Hai T, Bolouri H, Aderem A. 2006. Systems biology approaches identify ATF3 as a negative regulator of Toll-like receptor 4. Nature 441:173–178. doi:10.1038/nature04768. 16688168

[14] Chen S, Zuo X, Yang M, Lu H, Wang N, Wang K, Tu Z, Chen G, Liu M, Liu K, Xiao X. 2012. Severe multiple organ injury in HSF1 knockout mice induced by lipopolysaccharide is associated with an increase in neutrophil infiltration and surface expression of adhesion molecules. J Leukoc Biol 92:851–857. doi:10.1189/jlb.0212060. 22753951

[15] Shang L, Wang L, Shi X, Wang N, Zhao L, Wang J, Liu C. 2020. HMGB1 was negatively regulated by HSF1 and mediated the TLR4/MyD88/NF-κB signal pathway in asthma. Life Sci 241:117120. doi:10.1016/j.lfs.2019.117120. 31825792

[16] Kusumoto H, Hirohashi Y, Nishizawa S, Yamashita M, Yasuda K, Murai A, Takaya A, Mori T, Kubo T, Nakatsugawa M, Kanaseki T, Tsukahara T, Kondo T, Sato N, Hara I, Torigoe T. 2018. Cellular stress induces cancer stem-like cells through expression of DNAJB8 by activation of heat shock factor 1. Cancer Sci 109:741–750. doi:10.1111/cas.13501. 29316077 PMC5834799

[17] Schulze RJ, Krueger EW, Weller SG, Johnson KM, Casey CA, Schott MB, McNiven MA. 2020. Direct lysosome-based autophagy of lipid droplets in hepatocytes. Proc Natl Acad Sci USA 117:32443–32452. doi:10.1073/pnas.2011442117. 33288726 PMC7768785

[18] Deretic V, Levine B. 2018. Autophagy balances inflammation in innate immunity. Autophagy 14:243–251. doi:10.1080/15548627.2017.1402992. 29165043 PMC5902214

[19] Clarke AJ, Simon AK. 2019. Autophagy in the renewal, differentiation and homeostasis of immune cells. Nat Rev Immunol 19:170–183. doi:10.1038/s41577-018-0095-2. 30531943

[20] Finethy R, Dockterman J, Kutsch M, Orench-Rivera N, Wallace GD, Piro AS, Luoma S, Haldar AK, Hwang S, Martinez J, Kuehn MJ, Taylor GA, Coers J. 2020. Dynamin-related Irgm proteins modulate LPS-induced caspase-11 activation and septic shock. EMBO Rep 21:e50830. doi:10.15252/embr.202050830. 33124745 PMC7645254

[21] Yang T, Ren C, Lu C, Qiao P, Han X, Wang L, Wang D, Lv S, Sun Y, Yu Z. 2019. Phosphorylation of HSF1 by PIM2 induces PD-L1 expression and promotes tumor growth in breast cancer. Cancer Res 79:5233–5244. doi:10.1158/0008-5472.CAN-19-0063. 31409638

[22] Summers CM, Valentine RJ. 2019. Acute heat exposure alters autophagy signaling in C2C12 myotubes. Front Physiol 10:1521. doi:10.3389/fphys.2019.01521. 31969827 PMC6960406

[23] Vihervaara A, Sistonen L. 2014. HSF1 at a glance. J Cell Sci 127:261–266. doi:10.1242/jcs.132605. 24421309

[24] Desai S, Liu Z, Yao J, Patel N, Chen J, Wu Y, Ahn EE, Fodstad O, Tan M. 2013. Heat shock factor 1 (HSF1) controls chemoresistance and autophagy through transcriptional regulation of autophagy-related protein 7 (ATG7). J Biol Chem 288:9165–9176. doi:10.1074/jbc.M112.422071. 23386620 PMC3610989

[25] Zhang N, Wu Y, Lyu X, Li B, Yan X, Xiong H, Li X, Huang G, Zeng Y, Zhang Y, Lian J, Ni Z, He F. 2017. HSF1 upregulates ATG4B expression and enhances epirubicin-induced protective autophagy in hepatocellular carcinoma cells. Cancer Lett 409:81–90. doi:10.1016/j.canlet.2017.08.039. 28889000

[26] Samarasinghe B, Wales CT, Taylor FR, Jacobs AT. 2014. Heat shock factor 1 confers resistance to Hsp90 inhibitors through p62/SQSTM1 expression and promotion of autophagic flux. Biochem Pharmacol 87:445–455. doi:10.1016/j.bcp.2013.11.014. 24291777 PMC3934577

[27] Du P, Chang Y, Dai F, Wei C, Zhang Q, Li J. 2018. Role of heat shock transcription factor 1(HSF1)-upregulated macrophage in ameliorating pressure overload-induced heart failure in mice. Gene 667:10–17. doi:10.1016/j.gene.2018.04.042. 29678661

[28] Klionsky DJ, Abdel-Aziz AK, Abdelfatah S, Abdellatif M, Abdoli A, Abel S, Abeliovich H, Abildgaard MH, Abudu YP, Acevedo-Arozena A, Adamopoulos IE, Adeli K, Adolph TE, Adornetto A, Aflaki E, Agam G, Agarwal A, Aggarwal BB, Agnello M, Agostinis P, Agrewala JN, Agrotis A, Aguilar PV, Ahmad ST, Ahmed ZM, Ahumada-Castro U, Aits S, Aizawa S, Akkoc Y, Akoumianaki T, Akpinar HA, Al-Abd AM, Al-Akra L, Al-Gharaibeh A, Alaoui-Jamali MA, Alberti S, Alcocer-Gómez E, Alessandri C, Ali M, Alim Al-Bari MA, Aliwaini S, Alizadeh J, Almacellas E, Almasan A, Alonso A, Alonso GD, Altan-Bonnet N, Altieri DC, Álvarez ÉM, Alves S, et al. 2021. Guidelines for the use and interpretation of assays for monitoring autophagy (4th edition) (1). Autophagy 17:1–382. doi:10.1080/15548627.2020.1797280. 33634751 PMC7996087

[29] Kaiser SE, Qiu Y, Coats JE, Mao K, Klionsky DJ, Schulman BA. 2013. Structures of Atg7-Atg3 and Atg7-Atg10 reveal noncanonical mechanisms of E2 recruitment by the autophagy E1. Autophagy 9:778–780. doi:10.4161/auto.23644. 23388412 PMC3669186

[30] Pang Y, Yamamoto H, Sakamoto H, Oku M, Mutungi JK, Sahani MH, Kurikawa Y, Kita K, Noda NN, Sakai Y, Jia H, Mizushima N. 2019. Evolution from covalent conjugation to non-covalent interaction in the ubiquitin-like ATG12 system. Nat Struct Mol Biol 26:289–296. doi:10.1038/s41594-019-0204-3. 30911187

[31] Shams Nooraei M, Noori-Zadeh A, Darabi S, Rajaei F, Golmohammadi Z, Abbaszadeh HA. 2018. Low level of autophagy-related gene 10 (ATG10) expression in the 6-hydroxydopamine rat model of Parkinson’s disease. Iran Biomed J 22:15–21. doi:10.22034/ibj.22.1.15. 28734275 PMC5712380

[32] Jo YK, Kim SC, Park IJ, Park SJ, Jin DH, Hong SW, Cho DH, Kim JC. 2012. Increased expression of ATG10 in colorectal cancer is associated with lymphovascular invasion and lymph node metastasis. PLoS One 7:e52705. doi:10.1371/journal.pone.0052705. 23285162 PMC3527592

[33] Jo YK, Roh SA, Lee H, Park NY, Choi ES, Oh JH, Park SJ, Shin JH, Suh YA, Lee EK, Cho DH, Kim JC. 2017. Polypyrimidine tract-binding protein 1-mediated down-regulation of ATG10 facilitates metastasis of colorectal cancer cells. Cancer Lett 385:21–27. doi:10.1016/j.canlet.2016.11.002. 27836735

[34] Sun W, Li J, Zhou L, Han J, Liu R, Zhang H, Ning T, Gao Z, Liu B, Chen X, Ba Y. 2020. The c-Myc/miR-27b-3p/ATG10 regulatory axis regulates chemoresistance in colorectal cancer. Theranostics 10:1981–1996. doi:10.7150/thno.37621. 32104496 PMC7019154

[35] Xiao X, Zuo X, Davis AA, McMillan DR, Curry BB, Richardson JA, Benjamin IJ. 1999. HSF1 is required for extra-embryonic development, postnatal growth and protection during inflammatory responses in mice. EMBO J 18:5943–5952. doi:10.1093/emboj/18.21.5943. 10545106 PMC1171660

[36] Mohamed DA, Mohamed NM, Abdelrahaman S. 2020. Histological and biochemical changes in adult male rat liver after spinal cord injury with evaluation of the role of granulocyte-colony stimulating factor. Ultrastruct Pathol 44:395–411. doi:10.1080/01913123.2020.1844829. 33280459

[37] Yousef N, Vigo G, Shankar-Aguilera S, De Luca D. 2020. Semiquantitative ultrasound assessment of lung aeration correlates with lung tissue inflammation. Ultrasound Med Biol 46:1258–1262. doi:10.1016/j.ultrasmedbio.2020.01.018. 32081586

[38] Zhang H, Zhang L, Yu F, Liu Y, Liang Q, Deng G, Chen G, Liu M, Xiao X. 2012. HSF1 is a transcriptional activator of IL-10 gene expression in RAW264.7 macrophages. Inflammation 35:1558–1566. doi:10.1007/s10753-012-9471-4. 22549481

